# Classification of Hand Grasp Kinetics and Types Using Movement-Related Cortical Potentials and EEG Rhythms

**DOI:** 10.1155/2017/7470864

**Published:** 2017-08-29

**Authors:** Mads Jochumsen, Cecilie Rovsing, Helene Rovsing, Imran Khan Niazi, Kim Dremstrup, Ernest Nlandu Kamavuako

**Affiliations:** ^1^Centre for Sensory-Motor Interaction, Department of Health Science and Technology, Aalborg University, Aalborg, Denmark; ^2^New Zealand College of Chiropractic, Auckland, New Zealand; ^3^Rehabilitation Research Institute, Auckland University of Technology (AUT), Auckland, New Zealand

## Abstract

Detection of single-trial movement intentions from EEG is paramount for brain-computer interfacing in neurorehabilitation. These movement intentions contain task-related information and if this is decoded, the neurorehabilitation could potentially be optimized. The aim of this study was to classify single-trial movement intentions associated with two levels of force and speed and three different grasp types using EEG rhythms and components of the movement-related cortical potential (MRCP) as features. The feature importance was used to estimate encoding of discriminative information. Two data sets were used. 29 healthy subjects executed and imagined different hand movements, while EEG was recorded over the contralateral sensorimotor cortex. The following features were extracted: delta, theta, mu/alpha, beta, and gamma rhythms, readiness potential, negative slope, and motor potential of the MRCP. Sequential forward selection was performed, and classification was performed using linear discriminant analysis and support vector machines. Limited classification accuracies were obtained from the EEG rhythms and MRCP-components: 0.48 ± 0.05 (grasp types), 0.41 ± 0.07 (kinetic profiles, motor execution), and 0.39 ± 0.08 (kinetic profiles, motor imagination). Delta activity contributed the most but all features provided discriminative information. These findings suggest that information from the entire EEG spectrum is needed to discriminate between task-related parameters from single-trial movement intentions.

## 1. Introduction

The detection of movement intentions is an essential part of a brain-computer interface (BCI) for motor rehabilitation after a stroke [1]. By detecting movement intentions from the ongoing EEG, it is possible to activate an electrical stimulator or rehabilitation robot [2, 3], so the elicited somatosensory feedback is paired with motor cortical activity. In this way, the requirement for Hebbian learning is fulfilled. The detection of movement intentions from EEG, specifically movement-related cortical potentials (MRCPs), has been thoroughly investigated and several techniques exist to detect executed and imaginary movements from healthy subjects and attempted movements from patients suffering from spinal cord injury or stroke [4–9]. Recent studies have been published where the efficacy of BCI interventions for neurorehabilitation has shown promising results [1, 2]. To improve BCI interventions, task variability can be introduced into the rehabilitation which maximizes the retention of relearned movements [10]. Some studies have shown that it is possible to decode different task-related parameters from the same limb such as movement direction, movement type, force, and speed [7, 11–15]; by decoding such parameters, variability may be introduced in the training. In these studies, a wide variety of signal processing techniques and features have been used. The features, as for the movement intention detection, have primarily been extracted from the time and frequency domain. The features include mean amplitude in different time windows, either chosen systematically or based on the underlying physiology/signal morphology [7, 15], and spectral power in frequency bands that are systematically chosen with a width of, for example, 1–5 Hz or from the physiological EEG rhythms [7, 14, 16]. Other types of features have also been used such as time-frequency representations [17]. The features are often selected in an exhaustive systematic way to identify the features (and best channels) that fit the individual subject; in this way, it is possible to account for the great intersubject variability [7]. In summary, these studies show that task-related parameters can be decoded from single-trial analysis using different features extracted from premovement EEG. However, information is lacking regarding the importance of the features and where the discriminative information arise from in the physiologically established measures of EEG and MRCPs, that is, delta, theta, mu/alpha, beta, and gamma rhythms, readiness potential, negative slope, and motor potential, respectively. It has previously been shown that the components of the MRCP are modulated by variations in force and speed [18], but it is not known how variations in, for example, grasp types affect MRCPs. Moreover, it is not known how these task-related parameters modulate the different EEG rhythms. As outlined, many different kinds of features from the time and frequency domain have been used to classify single-trial EEG traces to discriminate between task-related parameters, but using only the established EEG rhythms (not to be mixed with the event-related synchronization/desynchronization) and MRCP components as features have not been evaluated. By testing this, it may be possible to explain the importance of the different features and give an indication of where the discriminative information is encoded.

In the current study, it was investigated if using the established EEG rhythms and MRCP components, extracted from the premovement EEG, can be used as features to discriminate between different task-related parameters for hand movements. Moreover, the importance of each feature type was investigated. For this investigation, two previously published data sets [6, 7] were used, which enables a direct comparison with previous results.

## 2. Methods

In the following sections, the data collection will be outlined as well as the analysis used in the current study.

### 2.1. Subjects

29 healthy subjects participated; 14 subjects (7 women and 7 men: 24 ± 1 years old) performed different grasp types (motor execution, data set 1), while 15 subjects (12 women and 3 men: 27 ± 11 years old) performed the same grasp type but with variations in the level of force and speed (motor execution and imagination, data set 2). All subjects gave their written informed consent. All procedures were approved by the local ethical committee (number 20130081).

### 2.2. Experimental Setup

**Figure 1:**
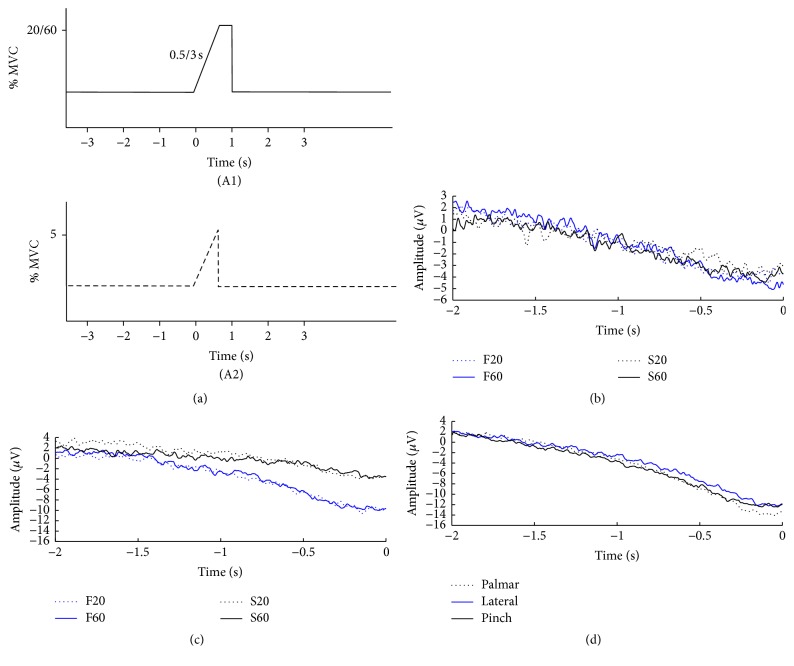
(a) Visual cues presented to the subjects performing movements with different kinetic profiles (A1) and grasp types (A2), (b) grand average across trials and subjects for imaginary movements with different kinetic profiles in channel C3, (c) grand average across trials and subjects for executed movements with different kinetic profiles in channel C3, and (d) grand average across trials and subjects for different executed grasp types in channel C3. F20: fast (0.5 s) 20% MVC, F60: fast (0.5 s) 60% MVC, S20: slow (3 s) 20% MVC, and S60: slow (3 s) 60% MVC. Note the difference in amplitude on the *y*-axis in (b). MVC: maximum voluntary contraction.

The subjects were seated in a comfortable chair with their right hand resting on a table in front of them. The subjects held a handgrip dynamometer which was used to record the force that was produced. The right hand was dominant in all subjects except for one. At the beginning of the experiment, the maximum voluntary contraction (MVC) was determined. In data set 1 [7], the subjects were asked to perform three different hand grasps: palmar, lateral, and pinch grasps, where they had to reach ~5% MVC in 0.5 s (see (A2) in Figure 1). Each movement type was performed 4 × 25 times with a 1-minute break in-between each every 25th movement. Two consecutive movements were separated with 9 s. The movements were performed in blocks; the order was randomized. The subjects were visually cued (see (A2) in Figure 1) by a custom-made program (Aalborg University), and the produced force was recorded and used as input, so the subjects had continuous visual feedback. The subjects spent ~5 minutes practicing to become familiar with the setup.

In data set 2 [6], the subjects were asked to execute and imagine four isometric palmar grasps. The tasks were as follows: 0.5 s to reach 20% MVC, 0.5 s to reach 60% MVC, 3 s to reach 20% MVC, and 3 s to reach 60% MVC; each task was repeated 40 times. The subjects were visually cued (see (A1) in Figure 1), and they were provided with visual feedback in the same way as described above to ensure that the movements were performed with the correct level of speed and force. No force was produced for the imagined movements, but the subjects were still provided with the feedback, so they knew when to initiate the imagined movement. The tasks were randomized in blocks, and the subjects trained for two minutes before each task.

### 2.3. Recordings

#### 2.3.1. EEG

Continuous monopolar (Ag/AgCl ring electrodes) EEG (EEG Amplifiers, Nuamps Express, Neuroscan) was recorded from the following channels (according to the International 10–20 system): F5, F3, F1, Fz, FC5, FC3, FC1, FCz, C5, C3, C1, Cz, CP5, CP3, CP1, CPz, P5, P3, P1, and Pz; moreover, F7, FT7, T7, TP7, and P7 for the subjects performing three different hand grasps (data set 1). The signals were referenced to the right ear lobe and grounded at nasion. Electrooculography (EOG) was recorded from FP1. The EEG and EOG were sampled with 500 Hz and converted with 32-bit precision. The impedance of all electrodes was below 5 k*Ω*. During the recordings, the subjects were asked to minimize eye blinks and facial and body movements. Epochs were rejected if they were contaminated with EOG, peak-peak amplitude exceeding 125 *μ*V. A digital trigger was sent from the visual cueing program to the EEG amplifier at the beginning of each trial (at *t* = −3 s in (A1) and (A2) in Figure 1).

#### 2.3.2. Force and Maximum Voluntary Contraction

A handgrip dynamometer (Noraxon USA, Scottsdale, AZ) was used to record the force, which was used as input to the visual cueing program. The force was sampled with 2000 Hz. The MVC was determined at the beginning of the experiment, where the subject performed three maximal contractions separated by one minute. The highest value of the three contractions was used as the MVC. For the tasks where the movements were executed, the force was used to determine the movement onset. This was defined as the instant where all values in a 200-ms wide moving time window were above the baseline. The baseline was calculated from the recordings during the rest phase. All onsets were visually inspected.

### 2.4. Signal Processing

#### 2.4.1. Preprocessing

Initially, the signals were bandpass filtered from 0.05 to 45 Hz using a 2nd order zero-phase digital Butterworth filter. For dataset 2, a large Laplacian spatial filter was applied to be able to compare the findings in the current study with the ones reported previously [6]. F7, F3, Fz, T7, C3, Cz, P7, P3, and Pz were used to calculate a surrogate channel with C3 as the central channel [6]. The continuous EEG was divided into epochs from the movement onset (or task onset for motor imagery) and 2 s prior this point. Epochs containing EOG activity in FP1 were rejected if the peak-peak amplitude was above 125 *μ*V.

#### 2.4.2. Feature Extraction

Features were extracted from the time domain and the frequency domain from the MRCP and natural EEG rhythms, respectively. Three time domain features were extracted: (1) average amplitude from −2 s to −0.5 s with respect to the movement onset (early contingent negative variation (CNV), early Bereitschaftspotential (BP), or readiness potential (RP)), (2) average amplitude from −0.5 to −0.15 s with respect to the movement onset (late CNV, late BP, or negative slope), and (3) the peak of maximum negativity (the motor potential). Five spectral features were extracted from the movement onset and 2 s prior to this point; these were the average power in the delta (0–4 Hz), theta (4–7 Hz), alpha (7–15 Hz), beta (15–30 Hz), and gamma (30–45 Hz) frequency range. The average power was calculated using power spectral density with a Hamming window. The time and frequency domain features were extracted from each channel from data set 1 and from the surrogate channel from data set 2. These features were extracted from single-trial EEG traces.

### 2.5. Feature Selection and Classification

The data were randomly divided into ten parts, where nine parts were used for training and the last was used for evaluation. On the training set sequential forward selection was performed [7]. The features were ranked by the separability of a 2-class problem, for example, palmar grasp versus rest (lateral and pinch grasp), based on *u*-statistics from Mann–Whitney's test. The features were ranked with the highest *u*-statistics first and the features with the lowest *u*-statistics in the end. With leave-one-out cross-validation on the training set, the classification accuracy was obtained with linear discriminant analysis using the feature with the highest *u*-statistics value. Then the feature with the 2nd highest *u*-statistics was included and the classification accuracy was calculated; if the classification accuracy improved, the feature was added to the candidate feature set; otherwise, it was discarded. This procedure was repeated until all features were evaluated. Since a 3-class and two 4-class problems were considered, the optimal features were evaluated for all pairwise comparisons (e.g., palmar versus rest, lateral versus rest, and pinch versus rest) after which 3 (or 4) candidate feature sets were obtained. The 3 (or 4) candidate feature sets were merged and another round of feature selection was performed to obtain the final feature set that was used for the classification of the test set.

After the feature selection, the test data were classified in two different ways according to the two data sets. For data set 1, linear discriminant analysis was performed on a 3-class problem. For data set 2, a support vector machine with a linear kernel was used to classify the features for the two 4-class problems. The two different classifiers were chosen, so it would be possible to compare the findings with the previous publications on the data sets where linear discriminant analysis [7] and support vector machines [6] were used. The classification of features extracted from data set 2 was performed in three ways: (1) without feature selection to be able to compare the results with previous findings, (2) with sequential forward selection to estimate the importance of each feature type, and (3) with principal component analysis (PCA). The number of principal components used was equal to the number of features selected by sequential forward selection. The average classification accuracy was calculated across the ten testing folds. Moreover, to estimate if a global classifier can be used to classify new data, classification accuracies were calculated with leave-one-subject-out cross-validation; this was done on data set 2 to have a low dimensionality of the feature vector (eight features).

### 2.6. Feature Importance Evaluation

The importance of each feature type and channel location (for data set 1) was investigated. In this study, the feature importance is defined as how often each feature is selected in the training folds using sequential forward selection. The importance of each individual feature type (delta power, etc.) was merged across all channels for data set 1; this was done to investigate the effect of the feature type. The importance of each channel was evaluated by merging all feature types for the specific channel. The feature importance was averaged across the subjects for the two 4-class problems (executed and imaginary movement with different kinetic profiles) and the 3-class problem (different executed grasp types). The number of times the individual features were selected was divided by the total number of selected features to obtain the feature importance in percent. Moreover, the same analyses were performed for the best half of the subject (*n* = 7) based on classification accuracy.

### 2.7. Analysis Investigating the Effect of Gender, Age, and Motor Execution versus Imagination

**Figure 2:**
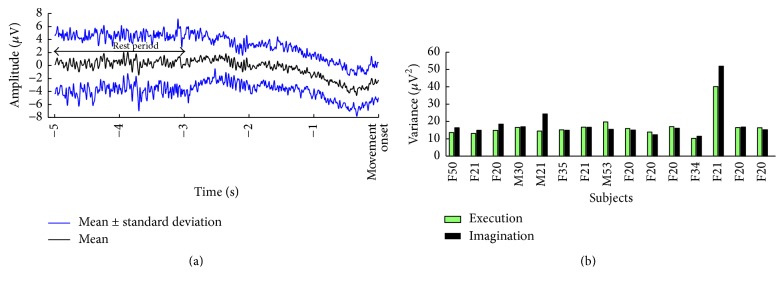
(a) Plot of the mean ± standard deviation of a representative subject (*n* = 1) performing motor execution to reach 60% MVC in 0.5 s. (b) The variance of the rest period is shown for each subject in data set 2. “M”: male, “F”: female, and the number is the age of the subject.

To investigate if the gender and age imbalance in data set 2 was affecting the results, an analysis was performed on the resting EEG for motor execution and imagination. Epochs were extracted from −5 to −3 s prior to the movement onset from the preprocessed EEG. The variance in the interval was calculated and plotted (see Figure 2) as well as the mean ± the standard deviation of the single-trial EEG −5 s until the movement onset.

## 3. Results

**Figure 3:**
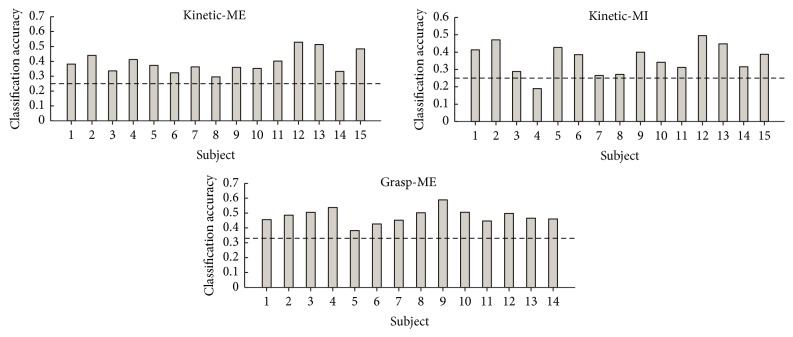
Individual classification accuracies across movement types for each subject. All classification accuracies are obtained after sequential forward selection. The theoretical chance levels have been added as horizontal dashed black lines.

**Figure 4:**
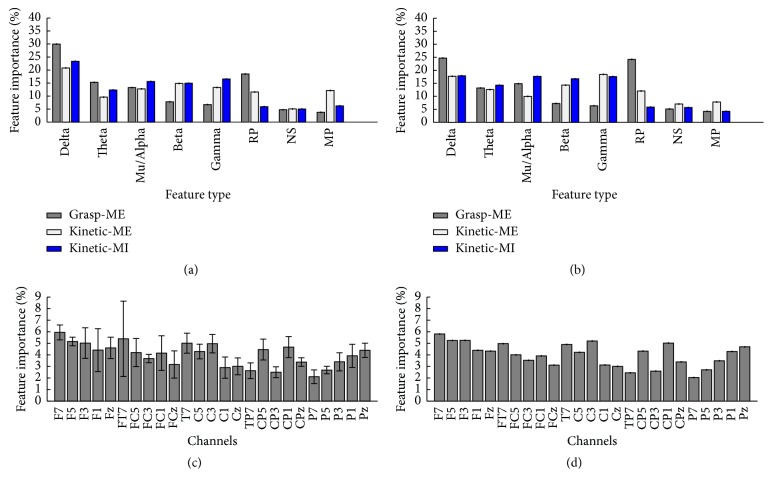
(a) Importance of each feature type for all subjects, (b) importance of each feature type for the seven best subjects (in terms of classification accuracy), (c) importance of each of the 25 channels in data set 1 for all subjects, and (d) importance of each of the 25 channels in data set 1 for the seven best subjects (in terms of classification accuracy). RP: readiness potential or early CNV/BP, NS: negative slope or late CNV/BP, and PN: peak negativity. The bars indicate ±1 × standard deviation.

From Figure 2(b), it can be seen that there is no trend for any differences related to gender or age, and the rest period for motor execution and imagination was similar. The classification accuracies are summarized in Tables 1–4 and in Figure 3, and the feature analysis is summarized in Figure 4. To investigate if there was an association between the ability to produce the specific force pattern and the classification accuracies, the root-mean-square error (RMSE) was calculated between the produced force and visual cue. The Spearman correlation (Rho: 0.25; *P* = 0.38) was calculated between the RMSE (0.25 ± 0.04) and the classification accuracies, but there was no association between the RMSE and the classification accuracies.

### 3.1. Classification of Movements

The results from the classification of the different grasp types (Table 1) show that the highest classification accuracies are on the diagonal; however, it should be noted that there is also a high number of misclassified samples. The overall classification accuracy for the 3-class problem was 0.48 ± 0.05 (mean ± standard deviation).

The results from the classification of the movements with different kinetics profiles (Tables 2 and 3) show that the highest classification accuracies are on the diagonal. Again, it should be noted that there is a high number of misclassified samples. The overall classification accuracies for the two 4-class problems were 0.41 ± 0.07 and 0.39 ± 0.08 (mean ± standard deviation) for motor execution and motor imagination, respectively, without using sequential forward selection. When the sequential forward selection was used, the classification accuracies were 0.39 ± 0.07 and 0.36 ± 0.09 for movement execution and motor imagination, respectively. For PCA, the classification accuracies were 0.38 ± 0.07 and 0.33 ± 0.09 for movement execution and motor imagination, respectively. In Figure 3, the intersubject variability in the classification accuracies is indicated.

In Table 4, the results are presented when using the leave-one-subject-out approach for estimating a global classifier where no training data are needed for the individual subject. With this approach, the average classification accuracies were 0.32 ± 0.04 and 0.31 ± 0.06 for movement execution and motor imagination, respectively. However, it should be noted that the highest values were only on the diagonal for fast 20% MVC and slow 60% MVC for motor execution and fast 20% MVC for motor imagination.

### 3.2. Feature and Channel Importance

The importance of each channel and feature type is outlined in Figure 4. No clear trend can be seen from the importance of each channel. The most important (most selected) feature type was the average power in the delta frequency range. The EEG rhythms were most important when discriminating between the movements with different kinetic profiles, but in general all of the eight feature types contain discriminative information. From Figure 4(a), it can be seen that the standard deviation of the feature importance across subjects is great. The patterns do not change much when only looking at the seven best subjects. There is a slight reduction in the importance of the delta activity and an increase in the importance of RP for the different grasp types.

## 4. Discussion

The results indicate that it is possible to discriminate between different grasp types and movements with different kinetic profiles, although the classification performance is limited. The most discriminative feature type was the power in the delta frequency range, but all of the features contributed discriminative information.

The classification accuracies obtained using the EEG rhythms and the MRCP segments were higher than chance level calculated with a significance level of 5% [19] when using the subject's own training data. The classification accuracies associated with the leave-one-subject-out approach were at chance level, which suggests that the classifier should be trained on the subject's own data. The classification accuracies were slightly higher for motor execution compared to motor imagination, which was also expected based on the signal morphology in Figure 1. This is also consistent with previous studies using temporal and spectral features [6, 16, 20]. The classification accuracies associated with the different grasp types were lower compared to previous findings [7]; however, it should be noted that the features were different, since the aim of the current study was to investigate where the discriminative information is encoded in the established EEG rhythms and MRCP components. The classification accuracies associated with the movements with different kinetic profiles were ~10 percentage points higher than in the reference study on data set 1 [6]. In the current study, extra features were added in terms of the average power of the EEG rhythms, and based on the analysis of the feature importance, the increase in classification accuracies is possibly due to the inclusion of those features.

The feature analysis revealed that task-related discriminative information can be extracted from the frequency range of all the different EEG rhythms with the main contribution from the delta band, which is also the frequency area where the MRCP is located. These findings are consistent with previous studies where it has been found that the entire EEG spectrum is used for discriminating between task-related parameters and that it is possible to decode the MRCP for different levels of force and speed [7, 15, 20]. It was, however, expected that the late BP/CNV and peak negativity would contribute more to the classification since they, according to the signal morphology, contain more discriminative information around the movement onset, at least for motor execution with different kinetic profiles (Figure 1(c)). Also, it has been shown that these segments were different for movements with different kinetic profiles [18, 21]. The single-trial variability (Figure 2(a)) may be an explanation for the fact that peak negativity is not so important for the classification or the relatively high cut-off frequency of the low pass filter when performing MRCP analysis; this should be around 5–10 Hz instead of 45 Hz if looking at the MRCP frequency range instead of the entire EEG spectrum. It should be noted that the RP and NS were extracted in fixed time intervals with respect to the movement onset to account for the single-trial variability; this has been done in several other studies [18, 22]. However, the different phases of the MRCP are affected by variations in, for example, attention [23], and the peak of maximum negativity may not always occur at the movement onset; therefore, the different phases could have been calculated with respect to the peak of maximum negativity instead of the movement onset. However, it may be difficult to identify the onset of the different phases (e.g., by changes in the slopes) in single-trial MRCPs in an automated way to avoid bias.

As well as the feature types, the importance of each channel was evaluated on data set 1. The analysis showed that all channels contributed discriminative information, which may be due to the size of the cortical representation of the hand and the effect of volume conduction. On average, the frontal channels contributed slightly more discriminative information which can be explained by the neural generation of the initial negative phase of the MRCP that is produced more frontally and then propagates more posteriorly. From a BCI control perspective, decoding of movement intentions is highly relevant; however, the performance is limited. It is not known what the lower limit of a BCI for rehabilitation is [24], but it is expected that the rehabilitative outcome is related to the BCI performance [2]. The performance could be increased by reducing the number of classes and focusing on two classes instead of four or by calibrating the BCI to the individual subject from a larger number of features (e.g., power from 1 Hz bins or wavelet analysis from each channel). This leads to a larger feature vector than what was reported in this study, whose focus was on established physiological features of the EEG and MRCP. The dimensionality of the large feature vector should therefore be reduced. Sequential forward selection and PCA showed similar performance; however, it is expected that PCA will perform worse when a larger number of features are included than the nine that were used in this study, but it will be much faster to compute the PCA [7].

## 5. Conclusion

It was shown that the task-related parameters, force, speed, and grasp type, can be decoded using the established EEG rhythms and MRCP components; although the performance was limited, it was above chance level. The delta rhythm contributed the most, but all EEG rhythms and MRCP components contained discriminative information regarding different levels of force and speed and about the type of hand grasp.

## Figures and Tables

**Table 1:** Classification accuracies obtained for the three different grasps. Pal: palmar grasp, Lat: lateral grasp, and Pin: pinch grasp.

Grasp	Predicted		
	Pal	Lat	Pin
True			
Pal	0.43	0.28	0.29
Lat	0.25	0.47	0.28
Pin	0.23	0.24	0.52

**Table 2:** Classification accuracies obtained for different motor execution kinetic profiles. F20: fast 20% MVC, F60: fast 60% MVC, S20: slow 20% MVC, and S60: slow 60% MVC. SFS: sequential forward selection, and PCA: principal component analysis. The classification accuracies obtained without and with SFS and PCA are presented in the top and bottom part, respectively.

	Predicted			
	F20	F60	S20	S60
*Kinetic-ME without SFS*				
True				
F20	0.45	0.20	0.22	0.14
F60	0.23	0.41	0.18	0.18
S20	0.22	0.20	0.38	0.20
S60	0.26	0.22	0.11	0.41

*Kinetic-ME with SFS*				
True				
F20	0.39	0.23	0.22	0.17
F60	0.26	0.35	0.20	0.19
S20	0.20	0.18	0.37	0.24
S60	0.25	0.21	0.09	0.45

*Kinetic-ME with PCA*				
True				
F20	0.39	0.27	0.19	0.15
F60	0.26	0.40	0.19	0.15
S20	0.25	0.18	0.34	0.23
S60	0.31	0.19	0.12	0.38

**Table 3:** Classification accuracies obtained for different motor imagination kinetic profiles. F20: fast 20% MVC, F60: fast 60% MVC, S20: slow 20% MVC, and S60: slow 60% MVC. SFS: sequential forward selection, and PCA: principal component analysis. The classification accuracies obtained without and with SFS and PCA are presented in the top and bottom part, respectively.

	Predicted			
	F20	F60	S20	S60
*Kinetic-MI without SFS*				
True				
F20	0.48	0.19	0.19	0.15
F60	0.23	0.33	0.27	0.17
S20	0.20	0.22	0.38	0.19
S60	0.30	0.22	0.11	0.37

*Kinetic-MI with SFS*				
True				
F20	0.42	0.21	0.18	0.20
F60	0.24	0.33	0.25	0.18
S20	0.22	0.22	0.35	0.22
S60	0.34	0.22	0.10	0.35

*Kinetic-MI with PCA*				
True				
F20	0.40	0.21	0.17	0.22
F60	0.25	0.31	0.25	0.19
S20	0.22	0.24	0.35	0.19
S60	0.32	0.30	0.10	0.28

**Table 4:** Classification accuracies obtained for different motor execution (top) and imagination (bottom) kinetic profiles using leave-one-subject-out classification (global classifier). F20: fast 20% MVC, F60: fast 60% MVC, S20: slow 20% MVC, and S60: slow 60% MVC. MVC: maximum voluntary contraction.

	Predicted			
	F20	F60	S20	S60
*Kinetic-ME*				
True				
F20	0.50	0.08	0.17	0.25
F60	0.49	0.09	0.21	0.21
S20	0.34	0.07	0.29	0.30
S60	0.35	0.06	0.17	0.41

*Kinetic-MI*				
True				
F20	0.55	0.05	0.22	0.18
F60	0.50	0.08	0.20	0.23
S20	0.44	0.05	0.28	0.22
S60	0.54	0.04	0.08	0.34
